# Insights on the Anti-Inflammatory and Anti-Melanogenic Effects of 2′-Hydroxy-2,6′-dimethoxychalcone in RAW 264.7 and B16F10 Cells

**DOI:** 10.3390/cimb47020085

**Published:** 2025-01-29

**Authors:** Sung-Min Bae, Chang-Gu Hyun

**Affiliations:** Department of Chemistry and Cosmetics, Jeju Inside Agency and Cosmetic Science Center, Jeju National University, Jeju 63243, Republic of Korea; 9901bae99@naver.com

**Keywords:** chalcone, cAMP/PKA, inflammation, MAPK, melanogenesis, NF-κB, PI3K/AKT, Wnt/β-catenin

## Abstract

Chalcones are recognized for their diverse pharmacological properties, including anti-inflammatory and anti-melanogenic effects. However, studies on 2′-hydroxy-2-methoxychalcone derivatives remain limited. This study investigated the anti-inflammatory and melanin synthesis-inhibitory effects of three derivatives: 2′-hydroxy-2,4-dimethoxychalcone (2,4-DMC), 2′-hydroxy-2,5′-dimethoxychalcone (2,5′-DMC), and 2′-hydroxy-2,6′-dimethoxychalcone (2,6′-DMC). In lipopolysaccharide (LPS)-stimulated RAW 264.7 macrophages, 2,6′-DMC demonstrated a superior inhibition of nitric oxide (NO) production, pro-inflammatory cytokines, and the expression of inducible nitric oxide synthase (iNOS) and cyclooxygenase-2 (COX-2) compared to the other derivatives. A mechanistic analysis revealed that 2,6′-DMC modulates the NF-κB and MAPK signaling pathways to attenuate inflammation. Additionally, 2,6′-DMC exhibited a significant inhibition of α-melanocyte-stimulating hormone (α-MSH)-induced melanin synthesis in B16F10 melanoma cells by downregulating tyrosinase, TRP-1, TRP-2, and MITF expression. This regulation was achieved through the suppression of the Wnt/β-catenin, PI3K/AKT, MAPK, and PKA/CREB pathways. Compared to 2,4-DMC and 2,5′-DMC, 2,6′-DMC’s structural configuration, characterized by methoxy groups at the 2- and 6′-positions, contributed to its enhanced molecular stability and binding affinity, amplifying its inhibitory effects. A primary skin irritation test confirmed that 2,6′-DMC exhibited minimal irritation, demonstrating its safety for dermal applications. These findings suggest that 2,6′-DMC holds promise as a dual-function agent for managing inflammatory conditions and hyperpigmentation-related disorders.

## 1. Introduction

Chalcones are a class of compounds characterized by a unique structural framework consisting of an α, β-unsaturated carbonyl system formed by the condensation of an aromatic aldehyde (typically benzaldehyde or its derivatives) and an aromatic ketone (such as acetophenone or its derivatives) [1]. Chemically, chalcones function as biosynthetic intermediates in the flavonoid biosynthesis pathway, a class of natural compounds abundantly present in various plants and fruits. Their distinctive structure—comprising two aromatic rings (A-ring and B-ring), an α, β-unsaturated double bond, and a carbonyl group—contributes to their high reactivity and broad range of pharmacological activities [2,3]. Additionally, the versatile nature of chalcone molecules allows for modifications at multiple positions on the aromatic rings, giving rise to a diverse array of derivatives with distinct chemical and biological properties [4].

Chalcone derivatives exhibit a broad spectrum of pharmacological activities, including antioxidant, anti-inflammatory, antimicrobial, anticancer, and melanin synthesis-inhibitory effects [5,6], indicating their potential as therapeutic or preventive agents for various diseases. Their beneficial effects are mediated through multiple physiological mechanisms. For instance, antioxidant activity can mitigate oxidative stress-related disorders, while anti-inflammatory properties are essential for managing inflammatory conditions [7]. Additionally, their anticancer effects highlight their potential to inhibit tumor growth, making them promising candidates for cancer therapy [8]. Recent studies also emphasize their therapeutic potential in addressing skin-related disorders, such as hyperpigmentation and UV-induced damage [9,10].

Inflammatory diseases remain a significant health concern globally as they encompass a wide range of conditions, such as inflammatory bowel disease, arthritis, and cardiovascular diseases, which severely impact quality of life [11,12]. The complex immune mechanisms involved in the onset and progression of these diseases underscore the need for safe and effective anti-inflammatory agents [13,14]. Inflammation is a critical defense mechanism, characterized by intricate interactions between immune cells and inflammatory mediators, including prostaglandins and cytokines. While an inflammatory response is essential for eliminating pathogens and repairing tissue damage, its dysregulation can lead to chronic inflammation and contribute to the development of persistent diseases [15,16].

Macrophages, which originate from blood monocytes, play a pivotal role in both the initiation and resolution of inflammation. Present in nearly all tissues, macrophages contribute to immune defense by engulfing pathogens, clearing cellular debris, and releasing the cytokines and chemokines that regulate an immune response and tissue repair [17,18]. RAW 264.7 macrophages, from a murine macrophage cell line, are frequently used as an experimental model in immunological and inflammatory research due to their similarity to primary macrophages in terms of cytokine production and inflammatory responses. This model system allows researchers to evaluate the efficacy and underlying mechanisms of novel anti-inflammatory compounds, thereby contributing to the development of new therapeutic strategies [19,20].

The skin, as the largest organ of the human body, serves a crucial protective role against external stressors, such as UV radiation and environmental pollutants [21]. A key component of this defense is melanin, which is produced by the melanocytes located in the basal layer of the epidermis. Melanin absorbs and dissipates UV radiation, thereby mitigating potential cellular damage. The synthesis of melanin begins with the activation of tyrosinase, a key enzyme that catalyzes the conversion of tyrosine into intermediate precursors [22]. However, when melanin is excessively produced or abnormally distributed—due to factors such as prolonged UV exposure, hormonal imbalances, inflammation, or aging—hyperpigmentation occurs. Common disorders associated with hyperpigmentation include freckles, melasma, and solar lentigines. Addressing these conditions requires the development of compounds that can effectively inhibit melanin synthesis without compromising skin health [23,24,25,26]. B16F10 melanoma cells, derived from murine melanocytes, are frequently used as a model system to study melanin biosynthesis and screen potential depigmenting agents. These cells provide valuable insights into the mechanisms of melanogenesis and support the discovery of treatments for both hyperpigmentation and melanoma [27,28].

Recent studies indicate that skin inflammation, often involving vascular dilation and tissue remodeling, is regulated by cytokines and chemokines, which influence melanocyte activation and melanin production. Elevated levels of inflammatory mediators can lead to abnormal pigmentation and hyperpigmentation-related disorders. Chronic inflammation, in particular, is strongly linked to skin pigmentary changes, emphasizing the need for therapeutic strategies that target both inflammation and pigmentation disorders [29,30,31].

**Figure 1:**
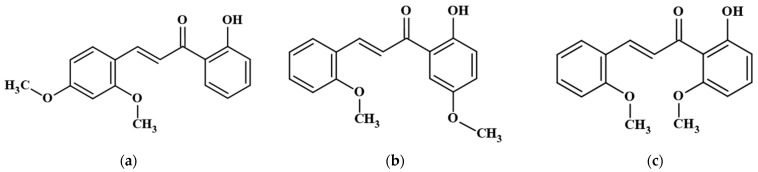
Structure of 2′-hydroxy-2-methoxychalcone derivatives. (**a**) 2′-hydroxy-2,4-dimethoxychalcone (2,4-DMC), (**b**) 2′-hydroxy-2,5′-dimethoxychalcone (2,5′-DMC), and (**c**) 2′-hydroxy-2,6′-dimethoxychalcone (2,6′-DMC).

Despite the extensive research on chalcone derivatives, studies focusing on 2′-hydroxy-2-methoxychalcone derivatives remain limited. In particular, the biophysiological effects of these compounds based on structural variations have yet to be fully elucidated. Therefore, this study aims to investigate the anti-inflammatory and melanin synthesis-inhibitory effects of 2′-hydroxy-2,4-dimethoxychalcone (2,4-DMC), 2′-hydroxy-2,5′-dimethoxychalcone (2,5′-DMC), and 2′-hydroxy-2,6′-dimethoxychalcone (2,6′-DMC) (Figure 1). By evaluating these derivatives, this study seeks to determine how structural differences influence their biological activities, contributing to the identification of potential therapeutic candidates for inflammatory and hyperpigmentation-related disorders.

## 2. Materials and Methods

### 2.1. Cell Culture

RAW 264.7 macrophage cells were obtained from the Korean Cell Line Bank (KCLB, Seoul, Republic of Korea), and B16F10 melanoma cells were sourced from the American Type Culture Collection (ATCC, Manassas, VA, USA). Both cell lines were maintained in Dulbecco’s Modified Eagle’s Medium (DMEM; Thermo Fisher Scientific, Waltham, MA, USA) supplemented with 10% heat-inactivated fetal bovine serum (FBS; Merck Millipore, Burlington, MA, USA) and 1% penicillin-streptomycin (Thermo Fisher Scientific). The cells were cultured at 37 °C in a humidified incubator with 5% CO_2_ (NB-203XL; N-BIOTEK, Inc., Bucheon, Republic of Korea). Cells between passages 10 and 15 were used for all experiments to ensure consistency and reproducibility.

### 2.2. Cell Viability Assessment by MTT Assay

The MTT assay was used to assess cell viability. RAW 264.7 cells were seeded at a density of 1.5 × 10^5^ cells/well in 24-well plates and incubated for 24 h. The cells were then treated with various concentrations (2.5, 5, 10, 20, and 40 μM) of the test compounds in the presence of LPS and incubated for an additional 24 h. Following treatment, the culture medium was replaced with 400 μL of 0.2 mg/mL MTT solution (diluted in a culture medium) and incubated for 3 h at 37 °C in a 5% CO_2_ atmosphere. The MTT-containing medium was then removed, and 800 μL of dimethyl sulfoxide (DMSO; Biosesang, Seongnam, Gyeonggi-do, Republic of Korea) was added to each well to dissolve the formazan crystals. Absorbance was measured at 570 nm using a microplate reader (Epoch, BioTek, Winooski, VT, USA). For B16F10 cells, the seeding density was 8.0 × 10^3^ cells/well. Cells were incubated for 24 h before treatment with the same concentration range of test compounds. After a 72 h incubation period, the procedure was performed as described above, and absorbance was measured at 570 nm.

### 2.3. Nitric Oxide Measurement

Nitric oxide (NO) production was quantified by measuring nitrite (NO_2_^−^) levels in the cell culture supernatant using the Griess reaction. RAW 264.7 cells were seeded in 24-well plates at 1.5 × 10^5^ cells/well and pre-incubated for 24 h. Cells were treated with test samples (2.5, 5, 10, 20, and 40 μM) in the presence of LPS and incubated for 24 h. Following treatment, 100 μL of supernatant was mixed with an equal volume of Griess reagent (1% sulfanilamide, 0.1% N-(1-naphthyl)ethylenediamine, and 2.5% phosphoric acid) in a 96-well plate and allowed to react for 10 min at room temperature. Absorbance was measured at 540 nm, and NO concentration was calculated using a standard curve of sodium nitrite.

### 2.4. Measurement of PGE_2_ and Pro-Inflammatory Cytokines

The effects of 2,6′-DMC on the production of prostaglandin E_2_ (PGE_2_) and pro-inflammatory cytokines (IL-1β, IL-6, and TNF-α) were measured using an enzyme-linked immunosorbent assay (ELISA) kits. RAW 264.7 cells were seeded at 1.5 × 10^5^ cells/well in 24-well plates and pre-incubated for 24 h. Cells were then treated with 2,6′-DMC (1.25, 2.5, 5, and 10 μM) in the presence of LPS and incubated for 24 h. After incubation, the culture medium was collected and centrifuged at 15,000 rpm for 20 min to remove debris. The supernatants were used for ELISA assays (BD Biosciences, Franklin Lakes, NJ, USA) following the manufacturer’s protocols.

### 2.5. Measurement of Melanin Content

B16F10 cells were seeded in 60 mm cell culture dishes at a density of 7.0 × 10^4^ cells/well and pre-incubated for 24 h. Cells were treated with α-MSH (Merck KGaA, Darmstadt, Germany) and the test compounds (2.5, 5, 10, 20, and 40 μM) for 72 h. After treatment, the medium was removed, and cells were washed twice with 1× PBS. Cells were lysed with a radioimmunoprecipitation assay (RIPA) buffer containing 1% protease inhibitor cocktail for 15 min at 4 °C. The lysates were centrifuged at 15,000 rpm for 15 min, and the resulting pellets were dissolved in 250 μL of 1N NaOH containing 10% DMSO at 80 °C. Absorbance of the dissolved melanin was measured at 405 nm.

### 2.6. Measurement of Intracellular Tyrosinase Activity

For intracellular tyrosinase activity, B16F10 cells were seeded in 60 mm dishes at 7.0 × 10^4^ cells/well and incubated for 24 h. After treatment with α-MSH and the test compounds (2.5, 5, 10, 20, and 40 μM) for 72 h, cells were washed twice with PBS and lysed with an RIPA buffer at 4 °C for 15 min. The lysates were centrifuged, and the supernatant was collected. Protein concentrations were adjusted to 20 μg/mL using a BCA protein assay kit. A 20 μL aliquot of each sample was mixed with 80 μL of 2 mg/mL L-DOPA and incubated at 37 °C for 2 h. Tyrosinase activity was measured at 490 nm.

### 2.7. Western Blot Analysis

For Western blot analysis, RAW 264.7 cells were seeded at 6.0 × 10^5^ cells/well in 60 mm dishes, while B16F10 cells were seeded at 7.0 × 10^4^ cells/well. After treatment, cells were lysed, and protein concentrations were adjusted to 30 μg/mL. Samples were denatured with a Laemmli buffer and separated by SDS-PAGE, followed by a transfer to PVDF membranes. The membranes were blocked with 5% skim milk and incubated overnight with primary antibodies (1:1000 dilution). After washing, the membranes were incubated with secondary antibodies (1:1000 dilution) for 2 h at room temperature. Protein bands were visualized using enhanced chemiluminescence (ECL) and imaged using the ChemiDoc system (Vilber Lourmat, France). The primary antibodies used for Western blot experiments included tyrosinase (SC-20035), TRP-1 (SC-166857), TRP-2 (SC-74439), and MITF (SC-71588), which were purchased from Santa Cruz Biotechnology (Dallas, TX, USA). Antibodies such as p-ERK (9101S), ERK (9102S), p-p38 (9211S), p38 (9212S), p-JNK (9251S), JNK (9252S), p-PKA (5661S), PKA (4782S), p-AKT (9271S), AKT (9272S), p-GSK-3β (9322S), GSK-3β (5676S), p-β-catenin (9561S), β-catenin (25362S), p-IκBα (9246S), IκBα (4812S), p65 (4764S), lamin B (12586), β-actin (4967S), and secondary antibodies (anti-mouse and anti-rabbit) were obtained from Cell Signaling Technology (Danvers, MA, USA). Additionally, anti-iNOS (2982S) was purchased from Merck Millipore (Burlington, MA, USA), and anti-COX-2 was obtained from BD Biosciences (Franklin Lakes, NJ, USA).

### 2.8. Human Primary Skin Irritation Test

The human skin irritation test was conducted in accordance with the Declaration of Helsinki and approved by the Institutional Review Board (IRB, 1-220777-A-N-01-B-DICN23044) of Dermapro Ltd. Thirty healthy volunteers aged 20 to 60 years participated. The test site was the upper back of each participant. After cleansing with 70% ethanol, 20 μL of each test substance dissolved in squalene was applied under an occlusive patch for 24 h. Skin assessments were conducted 20 min and 24 h after patch removal. The primary skin irritation responses were scored based on Personal Care Products Council (PCPC) guidelines, and the irritation index was calculated. Skin reactions classified as +5 were excluded as they indicate potential allergic rather than irritant responses (Table 1 and Table 2).

**Table 1:** Classification criteria for primary skin irritation assessment.

Grade	Description of Clinical Observation
+1	Slight erythema
+2	Moderate erythema, possibly with barely perceptible edema at the margin, papules may be present
+3	Moderate erythema, with generalized edema
+4	Severe erythema with severe edema, with or without vesicles
+5	Severe reaction spread beyond the area of the patch

**Table 2:** Assessment criteria for primary skin reaction.

Range of Response	Judgment
0.00 ≤ R ≤ 0.87	None to slight
0.87 ≤ R ≤ 2.42	Mild
2.42 ≤ R ≤ 3.44	Moderate
3.44 ≤ R	Severe

### 2.9. Statistical Analysis

All experimental data are presented as the mean ± standard deviation (SD) from at least three independent replicates. Statistical analysis was conducted using one-way analysis of variance (ANOVA), followed by Student’s *t*-test to assess the differences between groups. Statistical significance was defined as follows: # *p* < 0.001 compared to the untreated control group, and * *p* < 0.1, ** *p* < 0.01, and *** *p* < 0.001 compared to the treated control group.

## 3. Results

### 3.1. Effects of 2′-Hydroxy-2-methoxychalcone Derivatives on Cell Viability and NO Production in RAW 264.7 Cells

**Figure 2:**
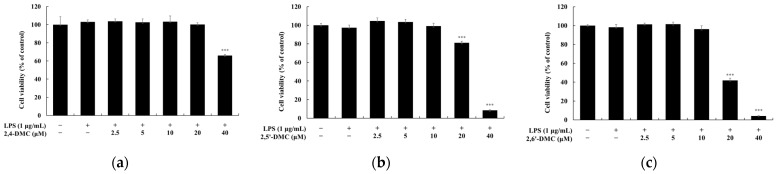
The effects of 2′-hydroxy-2-methoxychalcones on cell viability in LPS-stimulated RAW 264.7 cells. RAW 264.7 cells were treated with increasing concentrations (2.5, 5, 10, 20, and 40 μM) of 2′-hydroxy-2-methoxychalcone derivatives for 24 h in the presence of 1 μg/mL of LPS. Cell viability was measured using the MTT assay for (**a**) 2,4-DMC, (**b**) 2,5′-DMC, and (**c**) 2,6′-DMC. Data are expressed as mean ± SD from three independent experiments. Statistical significance relative to the untreated control group is indicated as *** *p* < 0.001.

**Figure 3:**
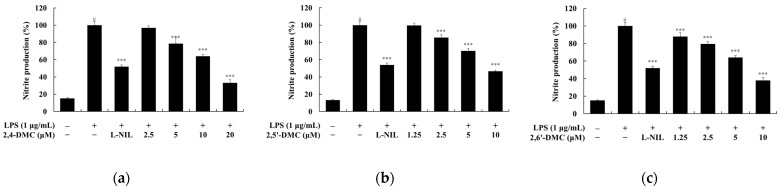
The effects of 2′-hydroxy-2-methoxychalcones on nitric oxide (NO) production in LPS-stimulated RAW 264.7 cells. RAW 264.7 cells were treated with 2′-hydroxy-2-methoxychalcone derivatives for 24 h following stimulation with 1 μg/mL of LPS. NO production was quantified using the Griess reagent for (**a**) 2,4-DMC, (**b**) 2,5′-DMC, and (**c**) 2,6′-DMC. Data are expressed as mean ± SD from three independent experiments. Statistical significance is indicated as # *p* < 0.001 compared to the untreated control group and *** *p* < 0.001 compared to the LPS-treated group.

To evaluate the cytotoxicity of 2′-hydroxy-2-methoxychalcone derivatives, an MTT assay was performed using RAW 264.7 macrophages treated with various concentrations of each compound (2.5–40 μM) for 24 h. If cell viability remained at or above 90% relative to the untreated control group, the treatment was considered non-cytotoxic. The results indicated that 2′-hydroxy-2,4-dimethoxychalcone (2,4-DMC) exhibited no cytotoxic effects at concentrations up to 20 μM, while 2′-hydroxy-2,5′-dimethoxychalcone (2,5′-DMC) and 2′-hydroxy-2,6′-dimethoxychalcone (2,6′-DMC) were non-cytotoxic at concentrations up to 10 μM (Figure 2). Subsequently, the effects of these compounds on nitric oxide (NO) production were investigated under non-cytotoxic conditions. After stimulation with 1 μg/mL lipopolysaccharide (LPS) to induce an inflammatory response, the cells were treated with varying concentrations of each compound for 24 h. L-NIL, a known iNOS inhibitor, was used as a positive control at 40 μM. The analysis revealed that at 10 μM, 2,6′-DMC reduced NO production by 61.95%, 2,5′-DMC by 53.22%, and 2,4-DMC by 36.04%, compared to the LPS-only treated group (Figure 3). These findings demonstrate that 2,6′-DMC exhibits the strongest inhibitory effect on NO production among the tested derivatives, highlighting its potential as an effective anti-inflammatory compound by suppressing NO synthesis in activated macrophages.

### 3.2. Effects of 2,6′-DMC on PGE_2_ and Pro-Inflammatory Cytokine Production in RAW 264.7 Cells

**Figure 4:**
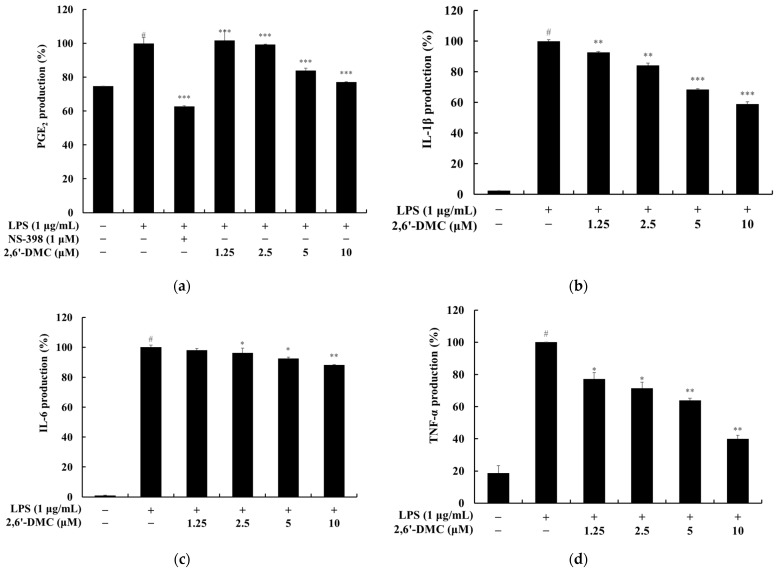
The effects of 2,6′-DMC on PGE_2_ and pro-inflammatory cytokine levels in LPS-stimulated RAW 264.7 cells. RAW 264.7 cells were treated with 2,6′-DMC for 24 h following stimulation with 1 μg/mL of LPS. (**a**) PGE_2_ levels, (**b**) IL-1β levels, (**c**) IL-6 levels, and (**d**) TNF-α levels were quantified using an ELISA kit. Data are presented as mean ± SD of three replicates. Statistical significance is indicated as # *p* < 0.001 compared to the untreated control group and * *p* < 0.1, ** *p* < 0.01, and *** *p* < 0.001 compared to the LPS-treated group.

The effects of 2,6′-DMC on the production of PGE_2_ and pro-inflammatory cytokines in LPS-stimulated RAW 264.7 macrophages were evaluated using an ELISA assay. In addition, NS-398, a selective cyclooxygenase-2 (COX-2) inhibitor, was used as a positive control. The results demonstrated a significant, dose-dependent reduction in the levels of PGE_2_ and pro-inflammatory cytokines, including IL-1β, IL-6, and TNF-α, following treatment with 2,6′-DMC. Notably, 2,6′-DMC effectively inhibited the expression of these inflammatory mediators in a concentration-dependent manner, indicating its potential to modulate key signaling pathways involved in inflammation (Figure 4).

These findings suggest that 2,6′-DMC attenuates inflammatory responses by suppressing the production of PGE_2_ and critical pro-inflammatory cytokines, supporting its potential role as an anti-inflammatory agent.

### 3.3. Effects of 2,6′-DMC on iNOS and COX-2 Expression in RAW 264.7 Cells

**Figure 5:**
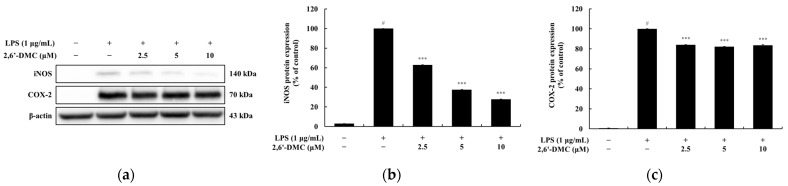
The effects of 2,6′-DMC on iNOS and COX-2 protein expression in LPS-stimulated RAW 264.7 cells. RAW 264.7 cells were treated with 2,6′-DMC for 24 h following stimulation with 1 μg/mL of LPS. (**a**) Representative Western blot images showing the expression of iNOS, and COX-2, with β-actin as a loading control. Quantification of (**b**) iNOS and (**c**) COX-2 protein expression levels. Data are presented as mean ± SD of three replicates. Statistical significance is indicated as # *p* < 0.001 compared to the untreated control group and *** *p* < 0.001 compared to the LPS-treated group.

A Western blot analysis was conducted to evaluate the effects of 2,6′-DMC on the expression levels of inducible nitric oxide synthase (iNOS) and COX-2 proteins in LPS-stimulated RAW 264.7 macrophages. The results demonstrated a significant, concentration-dependent reduction in the expression of both iNOS and COX-2. Specifically, treatment with 10 μM 2,6′-DMC decreased iNOS expression by 72.23% and COX-2 expression by 16.52% compared to the LPS-only treated control group (Figure 5). iNOS is a key enzyme responsible for the production of NO, a mediator of inflammatory responses, while COX-2 plays an essential role in the synthesis of prostaglandin E2 (PGE2), another pro-inflammatory molecule. The suppression of iNOS and COX-2 by 2,6′-DMC resulted in a corresponding decrease in NO and PGE2 levels. These findings suggest that 2,6′-DMC effectively inhibits the production of inflammatory mediators by downregulating iNOS and COX-2 expression, thereby contributing to the attenuation of inflammatory responses. This highlights the potential of 2,6′-DMC as a promising anti-inflammatory agent targeting key inflammatory enzymes.

### 3.4. Effects of 2,6′-DMC on the MAPK Pathway in RAW 264.7 Cells

**Figure 6:**
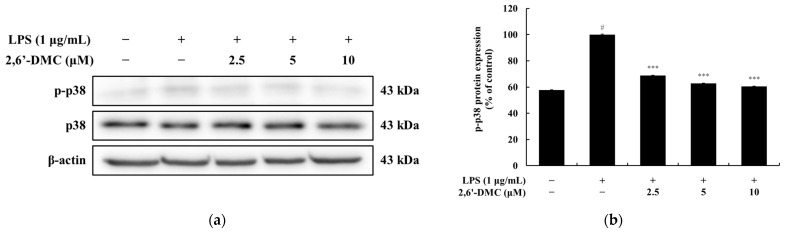
The effects of 2,6′-DMC on MAPK p38 phosphorylation in LPS-stimulated RAW 264.7 cells. RAW 264.7 cells were treated with 2,6′-DMC for 20 min following stimulation with 1 μg/mL of LPS. (**a**) Representative Western blot images showing the expression of phosphorylated p38 (p-p38), and total p38, with β-actin as a loading control. (**b**) Quantification of p-p38 protein expression levels. Data are presented as mean ± SD of three replicates. Statistical significance is indicated as # *p* < 0.001 compared to the untreated control group and *** *p* < 0.001 compared to the LPS-treated group.

A Western blot analysis was performed to examine the effects of 2,6′-DMC on the MAPK signaling pathway in LPS-stimulated RAW 264.7 macrophages. The results revealed a significant, dose-dependent reduction in the phosphorylation levels of p38, a key protein involved in the MAPK pathway. Specifically, treatment with 10 μM 2,6′-DMC decreased phosphorylated p38 levels by approximately 39.63% compared to the LPS-only treated control group (set at 100%) (Figure 6). These findings suggest that 2,6′-DMC effectively inhibits the phosphorylation of p38, a crucial mediator of inflammation, thereby contributing to the suppression of inflammatory responses. This study underscores the potential role of 2,6′-DMC as an anti-inflammatory agent through the modulation of the MAPK signaling pathway.

### 3.5. Effects of 2,6′-DMC on IκB-α Phosphorylation in RAW 264.7 Cells

**Figure 7:**
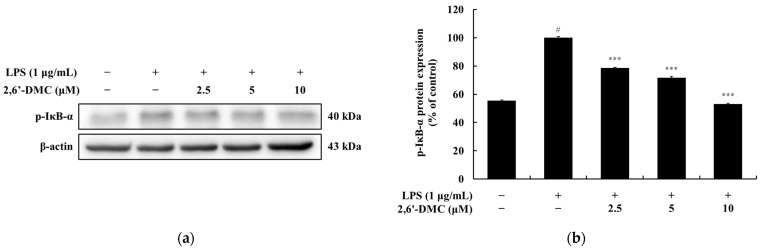
The effects of 2,6′-DMC on IκB-α phosphorylation in LPS-stimulated RAW 264.7 cells. RAW 264.7 cells were treated with 2,6′-DMC for 15 min following stimulation with 1 μg/mL of LPS. (**a**) Representative Western blot images showing the expression of phosphorylated IκB-α (p-IκB-α) with β-actin as a loading control. (**b**) Quantification of p-IκB-α protein expression levels. Data are presented as mean ± SD of three replicates. Statistical significance is indicated as # *p* < 0.001 compared to the untreated control group and *** *p* < 0.001 compared to the LPS-treated group.

A Western blot analysis was conducted to investigate the effects of 2,6′-DMC on the NF-κB signaling pathway in LPS-stimulated RAW 264.7 macrophages. The results demonstrated a significant dose-dependent reduction in the phosphorylation levels of IκB-α. Notably, treatment with 10 μM 2,6′-DMC decreased phosphorylated IκB-α levels by approximately 46.92% compared to the LPS-only treated control group (set at 100%) (Figure 7). Since the phosphorylation of IκB-α is a critical step in the activation of NF-κB, its reduction prevents the dissociation of NF-κB from IκB-α, thereby inhibiting NF-κB nuclear translocation. This suppression results in the downregulation of pro-inflammatory transcription factors and cytokines, highlighting the anti-inflammatory potential of 2,6′-DMC through the modulation of the NF-κB signaling pathway.

### 3.6. Effects of 2,6′-DMC on NF-κB Nuclear Translocation in RAW 264.7 Cells

**Figure 8:**
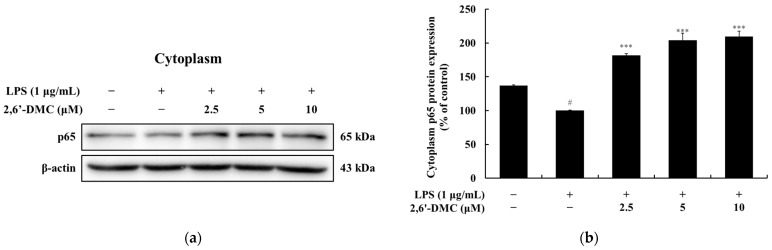
The effects of 2,6′-DMC on NF-κB p65 protein expression in LPS-stimulated RAW 264.7 cells. RAW 264.7 cells were treated with 2,6′-DMC for 15 min following stimulation with 1 μg/mL of LPS. (**a**) Representative Western blot images showing p65 expression in the cytoplasm with β-actin as a loading control. (**b**) Quantification of p65 protein expression levels in the cytoplasm. Statistical significance is indicated as # *p* < 0.001 compared to the untreated control group and *** *p* < 0.001 compared to the LPS-treated group.

A Western blot analysis was conducted to investigate the effects of 2,6′-DMC on p65 expression in LPS-stimulated RAW 264.7 macrophages. The results showed a significant, dose-dependent increase in p65 expression levels in the cytoplasm. Specifically, treatment with 10 μM 2,6′-DMC increased cytoplasmic p65 levels by approximately 109.35% compared to the LPS-only treatment group (Figure 8). The increase in cytoplasmic p65 expression suggests the potential of 2,6′-DMC as a pharmacological agent with anti-inflammatory activity through the modulation of the NF-κB signaling pathway.

### 3.7. Cytotoxicity Assessment of 2′-Hydroxy-2-methoxychalcone Derivatives in B16F10 Cells

**Figure 9:**
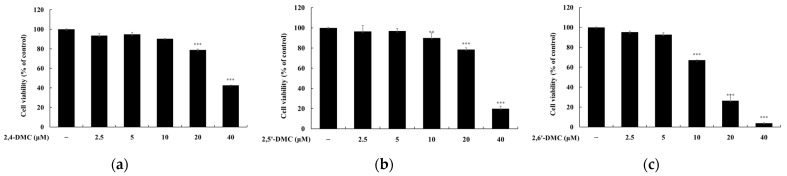
The effects of 2′-hydroxy-2-methoxychalcones on cell viability in B16F10 melanoma cells. B16F10 cells were treated with increasing concentrations (2.5, 5, 10, 20, and 40 μM) of 2′-hydroxy-2-methoxychalcone derivatives. Cell viability was assessed using the MTT assay for (**a**) 2,4-DMC, (**b**) 2,5′-DMC, and (**c**) 2,6′-DMC. Data are presented as mean ± SD from three independent experiments. Statistical significance relative to the untreated control group is indicated as ** *p* < 0.001 and *** *p* < 0.001.

An MTT assay was conducted to assess the effects of 2′-hydroxy-2-methoxychalcone derivatives on the viability of B16F10 melanoma cells. Cells were treated with various concentrations of each compound (2.5–40 μM) and incubated for 72 h. Cell viability ≥90% relative to the untreated control group was considered indicative of no cytotoxic effects. The results demonstrated that 2′-hydroxy-2,4-dimethoxychalcone (2,4-DMC) did not exhibit cytotoxicity at concentrations up to 10 μM. In contrast, 2′-hydroxy-2,5′-dimethoxychalcone (2,5′-DMC) and 2′-hydroxy-2,6′-dimethoxychalcone (2,6′-DMC) were non-cytotoxic at concentrations up to 5 μM. These findings indicate that 2,4-DMC has a broader non-cytotoxic concentration range compared to 2,5′-DMC and 2,6′-DMC (Figure 9). This suggests that appropriate concentration ranges should be carefully considered when using these derivatives in subsequent studies to ensure cellular viability while investigating their biological activities.

### 3.8. Effects of 2′-Hydroxy-2-methoxychalcone Derivatives on Melanin Synthesis and Tyrosinase Activity in B16F10 Cells

**Figure 10:**
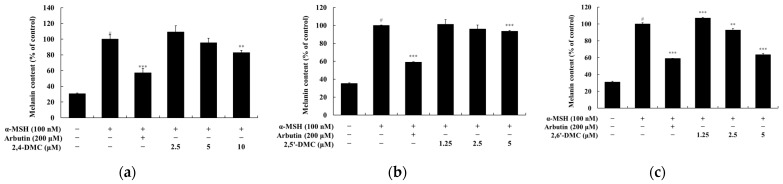
The effects of 2′-hydroxy-2-methoxychalcones on melanin content in α-MSH-stimulated B16F10 melanoma cells. B16F10 cells were treated with 2′-hydroxy-2-methoxychalcone derivatives for 72 h following stimulation with α-MSH. Melanin content is displayed as follows: (**a**) 2,4-DMC, (**b**) 2,5′-DMC, and (**c**) 2,6′-DMC. Data are presented as mean ± SD from three independent experiments. Statistical significance is indicated as # *p* < 0.001 compared to the untreated control group and ** *p* < 0.01, and *** *p* < 0.001 compared to the α-MSH-treated group.

**Figure 11:**
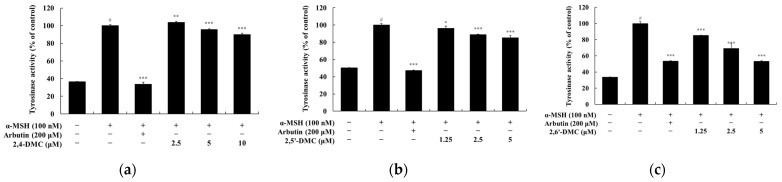
The effects of 2′-hydroxy-2-methoxychalcones on tyrosinase activity in α-MSH-stimulated B16F10 melanoma cells. B16F10 cells were treated with 2′-hydroxy-2-methoxychalcone derivatives for 72 h following stimulation with α-MSH. Tyrosinase activity is shown in the graphs as follows: (**a**) 2,4-DMC, (**b**) 2,5′-DMC, and (**c**) 2,6′-DMC. Data are presented as mean ± SD from three independent experiments. Statistical significance is indicated as # *p* < 0.001 compared to the untreated control group and * *p* < 0.1, ** *p* < 0.01, and *** *p* < 0.001 compared to the α-MSH-treated group.

Melanin content and tyrosinase activity assays were conducted to evaluate the effects of 2′-hydroxy-2-methoxychalcone derivatives on melanin synthesis and tyrosinase activity in B16F10 melanoma cells at non-cytotoxic concentrations. B16F10 cells were treated with various concentrations of each compound and incubated for 72 h. α-MSH and arbutin were used as negative and positive controls, respectively. The results indicated that 2,6′-DMC at a concentration of 5 μM significantly inhibited melanin production by approximately 36.62% compared to the α-MSH-treated control group. In contrast, 2,5′-DMC and 2,4-DMC exhibited weaker inhibitory effects, reducing melanin content by approximately 6.65% and 4.68%, respectively (Figure 10). For tyrosinase activity, 2,6′-DMC showed an inhibitory effect of approximately 46.83% relative to the α-MSH-only treatment group. In comparison, 2,5′-DMC and 2,4-DMC showed inhibition rates of 14.69% and 4.23%, respectively (Figure 11). These findings highlight the superior inhibitory effects of 2,6′-DMC on both melanin synthesis and tyrosinase activity compared to the other derivatives, supporting its potential as an effective modulator of melanogenesis.

### 3.9. Effects of 2,6′-DMC on Melanin Synthesis Enzyme Expression in B16F10 Cells

**Figure 12:**
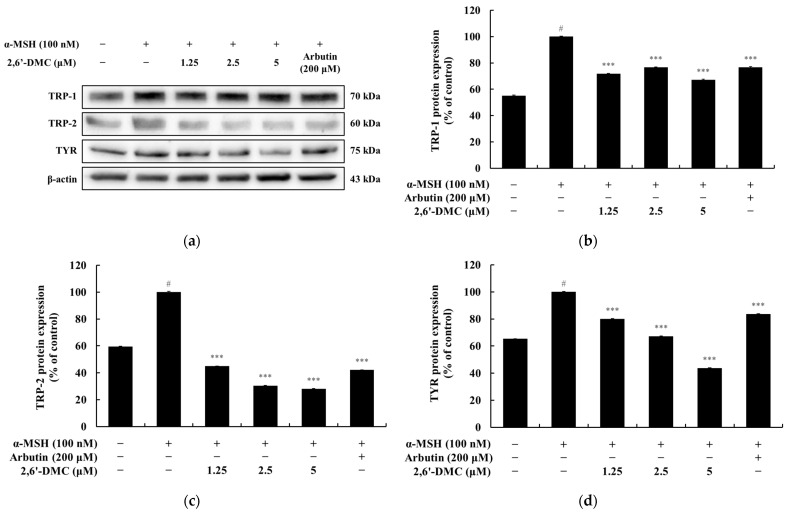
The effects of 2,6′-DMC on melanin synthesis enzyme expression in α-MSH-stimulated B16F10 melanoma cells. B16F10 cells were treated with 2,6′-DMC for 24 h following stimulation with α-MSH. (**a**) Representative Western blot images showing the expression of TRP-1, TRP-2, and TYR, with β-actin as a loading control. Quantification of (**b**) TRP-1, (**c**) TRP-2, and (**d**) TYR protein expression levels. Data are presented as mean ± SD of three replicates. Statistical significance is indicated as # *p* < 0.001 compared to the untreated control group and *** *p* < 0.001 compared to the α-MSH-treated group.

A Western blot analysis was conducted to assess the effects of 2,6′-DMC on the expression of the key enzymes involved in melanin synthesis in B16F10 melanoma cells. The results demonstrated a significant reduction in the expression levels of tyrosinase (TYR), tyrosinase-related protein 1 (TRP-1), and tyrosinase-related protein 2 (TRP-2) following 2,6′-DMC treatment. Specifically, treatment with 5 μM 2,6′-DMC decreased TYR, TRP-1, and TRP-2 levels by approximately 32.77%, 71.90%, and 56.16%, respectively, compared to the α-MSH-treated control group (Figure 12). These findings indicate that 2,6′-DMC suppresses the expression of critical melanogenic enzymes, contributing to a reduction in melanin synthesis. This highlights the potential role of 2,6′-DMC in modulating pigmentation by downregulating the enzymatic machinery essential for melanogenesis.

### 3.10. Effects of 2,6′-DMC on MITF Protein Expression in B16F10 Cells

**Figure 13:**
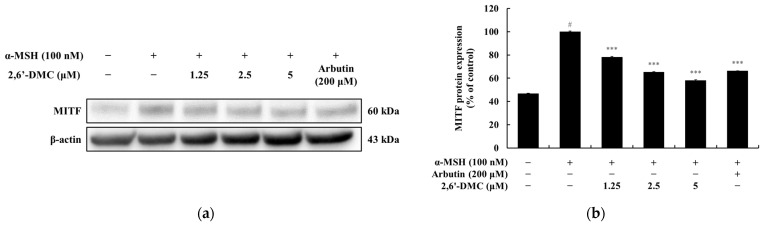
The effects of 2,6′-DMC on MITF protein expression in α-MSH-stimulated B16F10 melanoma cells. B16F10 cells were treated with 2,6′-DMC for 24 h following stimulation with α-MSH. (**a**) Representative Western blot images showing the expression of MITF with β-actin as a loading control. (**b**) Quantification of MITF protein expression levels. Data are presented as mean ± SD of three replicates. Statistical significance is indicated as # *p* < 0.001 compared to the untreated control group and *** *p* < 0.001 compared to the α-MSH-treated group.

A Western blot analysis was conducted to investigate the effects of 2,6′-DMC on MITF protein expression in B16F10 melanoma cells. The results indicated that 2,6′-DMC treatment significantly reduced MITF protein levels in a dose-dependent manner. Specifically, treatment with 5 μM 2,6′-DMC led to an approximately 41.76% reduction in MITF expression compared to the α-MSH-treated control group (Figure 13). These findings suggest that 2,6′-DMC effectively suppresses the expression of MITF, a key transcription factor involved in melanogenesis. The inhibition of MITF expression is likely to result in the downregulation of melanogenic enzymes, including tyrosinase, TRP-1, and TRP-2, ultimately leading to a reduction in melanin synthesis. This highlights the potential of 2,6′-DMC as a modulator of pigmentation by targeting the MITF-dependent regulatory pathway.

### 3.11. Effects of 2,6′-DMC on the Wnt/β-Catenin Signaling Pathway in B16F10 Cells

**Figure 14:**
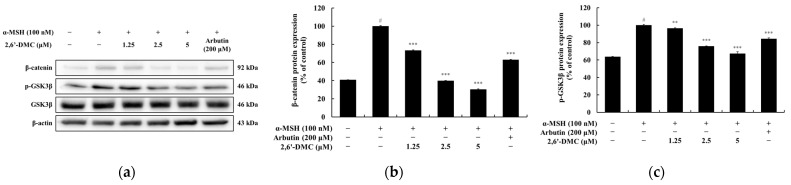
The effects of 2,6′-DMC on the Wnt/β-catenin signaling pathway in α-MSH-stimulated B16F10 melanoma cells. B16F10 cells were treated with 2,6′-DMC for 24 h following stimulation with α-MSH. (**a**) Representative Western blot images showing the expression levels of β-catenin, phosphorylated GSK3β (p-GSK3β), and total GSK3β, with β-actin as a loading control. Quantification of (**b**) β-catenin and (**c**) p-GSK3β protein expression levels. Data are presented as mean ± SD of three replicates. Statistical significance is indicated as # *p* < 0.001 compared to the untreated control group and ** *p* < 0.01, and *** *p* < 0.001 compared to the α-MSH-treated group.

A Western blot analysis was performed to evaluate the effects of 2,6′-DMC on the Wnt/β-catenin signaling pathway in B16F10 melanoma cells. The results demonstrated a significant reduction in the expression levels of β-catenin and the phosphorylation levels of GSK3β following 2,6′-DMC treatment. Specifically, treatment with 5 μM 2,6′-DMC decreased β-catenin expression and phosphorylated GSK3β levels by approximately 69.57% and 32.62%, respectively, compared to the α-MSH-treated control group (Figure 14). These findings indicate that 2,6′-DMC downregulates key proteins involved in the Wnt/β-catenin signaling pathway, contributing to the suppression of melanin synthesis. This suggests that the inhibitory effects of 2,6′-DMC on melanogenesis may be mediated through the modulation of the Wnt/β-catenin axis.

### 3.12. Effects of 2,6′-DMC on the PI3K/AKT Signaling Pathway in B16F10 Cells

**Figure 15:**
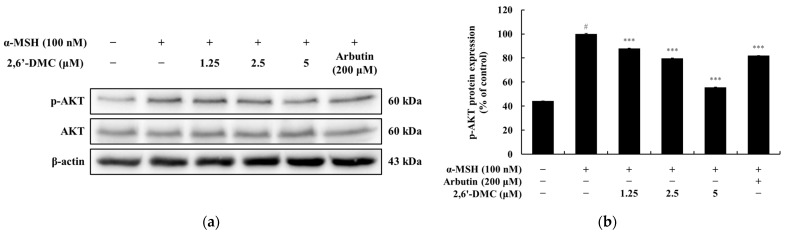
The effects of 2,6′-DMC on the PI3K/AKT signaling pathway in α-MSH-stimulated B16F10 melanoma cells. B16F10 cells were treated with 2,6′-DMC for 4 h following stimulation with α-MSH. (**a**) Representative Western blot images showing the expression levels of phosphorylated AKT (p-AKT), and total AKT, with β-actin as a loading control. (**b**) Quantitative analysis of p-AKT protein expression levels. Data are presented as mean ± SD of three replicates. Statistical significance is indicated as # *p* < 0.001 compared to the untreated control group and *** *p* < 0.001 compared to the α-MSH-treated group.

A Western blot analysis was conducted to examine the effects of 2,6′-DMC on the PI3K/AKT signaling pathway in B16F10 melanoma cells. The results indicated that 2,6′-DMC treatment significantly decreased the phosphorylation levels of AKT proteins in a dose-dependent manner. Notably, treatment with 5 μM 2,6′-DMC reduced phosphorylated AKT levels by approximately 44.36% compared to the α-MSH-treated control group (Figure 15).

These findings suggest that 2,6′-DMC inhibits the PI3K/AKT signaling pathway, which plays a crucial role in regulating the intracellular processes associated with melanin synthesis. This suppression of AKT phosphorylation highlights the potential mechanism by which 2,6′-DMC modulates melanogenesis.

### 3.13. Effects of 2,6′-DMC on the MAPK Signaling Pathway in B16F10 Cells

**Figure 16:**
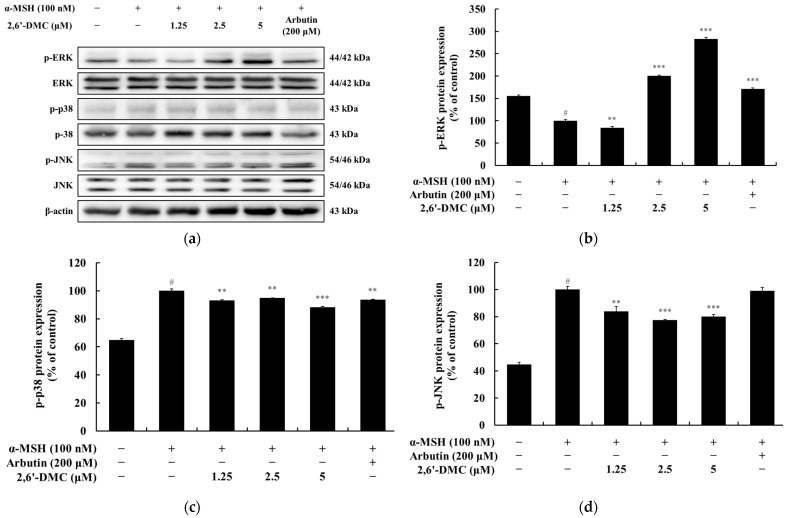
The effects of 2,6′-DMC on the MAPK signaling pathway in α-MSH-stimulated B16F10 cells. B16F10 cells were treated with 2,6′-DMC for 4 h following stimulation with α-MSH. (**a**) Representative Western blot images showing the expression levels of phosphorylated ERK (p-ERK), total ERK, phosphorylated p38 (p-p38), total p38, phosphorylated JNK (p-JNK), and total JNK, with β-actin as a loading control. Quantitative analysis of (**b**) p-ERK, (**c**) p-p38, and (**d**) p-JNK protein expression levels. Data are presented as mean ± SD of three replicates. Statistical significance is indicated as # *p* < 0.001 compared to the untreated control group and ** *p* < 0.01, and *** *p* < 0.001 compared to the α-MSH-treated group.

A Western blot analysis was conducted to evaluate the effects of 2,6′-DMC on the MAPK signaling pathway in B16F10 melanoma cells. The MAPK pathway, which regulates key cellular processes such as survival, differentiation, and stress responses, consists of ERK, p38, and JNK. The results demonstrated that treatment with 2,6′-DMC led to a significant increase in the phosphorylation level of ERK, while the phosphorylation levels of p38 and JNK were notably decreased. Specifically, treatment with 5 µM 2,6′-DMC increased phosphorylated ERK levels by approximately 182.74% compared to the α-MSH-treated control group, whereas phosphorylated p38 and JNK levels were reduced by 11.83% and 19.93%, respectively (Figure 16). These findings suggest that 2,6′-DMC modulates the MAPK signaling pathway by enhancing ERK activation and suppressing the activities of p38 and JNK. This regulatory effect contributes to the inhibition of melanin synthesis, highlighting the potential of 2,6′-DMC as a modulator of melanogenesis through MAPK pathway regulation.

### 3.14. Effects of 2,6′-DMC on the PKA/CREB Signaling Pathway in B16F10 Cells

**Figure 17:**
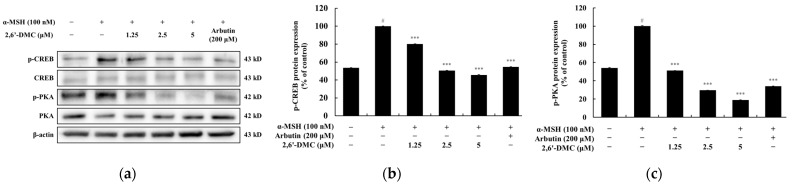
The effects of 2,6′-DMC on the PKA/CREB signaling pathway in α-MSH-stimulated B16F10 cells. B16F10 cells were treated with 2,6′-DMC for 4 h following stimulation with α-MSH. (**a**) Representative Western blot images showing the expression levels of phosphorylated PKA (p-PKA), total PKA, phosphorylated CREB (p-CREB), and total CREB, with β-actin as a loading control. Quantitative analysis of (**b**) p-PKA and (**c**) p-CREB protein expression levels. Data are presented as mean ± SD of three replicates. Statistical significance is indicated as # *p* < 0.001 compared to the untreated control group and *** *p* < 0.001 compared to the α-MSH-treated group.

To investigate the impact of 2,6′-DMC on the PKA/CREB signaling pathway in B16F10 cells, a Western blot analysis was performed. The results revealed a dose-dependent reduction in the phosphorylation levels of PKA and CREB following 2,6′-DMC treatment. Notably, in the cells treated with 5 μM 2,6′-DMC, the phosphorylation levels of CREB and PKA were reduced by approximately 54.31% and 81.06%, respectively, compared to the α-MSH-stimulated control group (Figure 17). These findings indicate that 2,6′-DMC suppresses the phosphorylation of PKA and CREB, which are critical regulators of melanogenesis. This suggests that 2,6′-DMC effectively modulates the melanin synthesis pathway by inhibiting upstream signaling events in the PKA/CREB axis.

### 3.15. Primary Skin Irritation Assessment of 2,6′-DMC

A clinical study was performed to evaluate the primary skin irritation potential of 2,6′-DMC on human skin, following the established ethical guidelines and inclusion/exclusion criteria. A total of 33 female volunteers participated in this study, with a mean age of 45.82 ± 7.84 years (age range: 25 to 53 years). All participants provided written informed consent prior to study enrollment. The findings demonstrated that 2,6′-DMC exhibits a low irritation potential. No significant adverse effects, such as persistent erythema or edema, were observed throughout the study period. Accordingly, 2,6′-DMC was classified as a low irritant in terms of primary skin irritation (Table 3). These results suggest that 2,6′-DMC is suitable for use in formulations where skin exposure occurs, supporting its potential application in cosmetic and dermatological products. Further investigations are recommended to assess its long-term safety and compatibility across diverse skin types.

**Table 3:** Skin irritation evaluation of 2,6′-DMC at different concentrations based on patch test reactions.

No	Samples	Responders	1st Assessment				2nd Assessment				Reaction Grade (R *)		
			+1	+2	+3	+4	+1	+2	+3	+4	20 min	24 h	Mean
1	2,6′-DMC (10 μM)	0	0	0	0	0	0	0	0	0	0	0	0
2	2,6′-DMC (5 μM)	0	0	0	0	0	0	0	0	0	0	0	0
3	Squalene (Control)	0	0	0	0	0	0	0	0	0	0	0	0

* Low irritation category (none to slight): 0.00 ≤ R < 0.87.

## 4. Discussion

The chalcone scaffold has garnered significant attention in medicinal chemistry due to its simple chemical structure, ease of synthesis for diverse derivatives, and ability to modulate multiple molecular targets, resulting in a broad range of pharmacological activities [1,2,3,4,5,6,7,8,9,10]. However, the biological effects of the structural variations among 2′-hydroxy-2-methoxychalcone derivatives, particularly their roles in modulating inflammation and melanogenesis, remain insufficiently studied. This study aimed to address this gap by analyzing the structural features of the selected 2′-hydroxy-2-methoxychalcone derivatives and evaluating their inhibitory effects on inflammatory responses in RAW 264.7 cells and melanin synthesis in B16F10 cells to elucidate their pharmacological potential.

Inflammation is a critical defense mechanism that protects the body from harmful stimuli and tissue injury. However, excessive or chronic inflammation can lead to tissue damage and contribute to the development of inflammatory diseases, such as arthritis, inflammatory bowel disease, and cardiovascular disorders [11,12,13,14,15,16]. While anti-inflammatory agents, such as steroids and non-steroidal anti-inflammatory drugs (NSAIDs), are effective, prolonged use is associated with adverse effects, including hepatotoxicity, gastrointestinal damage, and immunosuppression [32,33]. Therefore, the development of safer and more effective anti-inflammatory compounds is essential. In this context, the present study demonstrated that 2′-hydroxy-2-methoxychalcone derivatives suppressed the production of inflammatory mediators in RAW 264.7 cells (Figure 1).

To evaluate the anti-inflammatory effects, cell viability was measured using the MTT assay, and non-cytotoxic concentrations were used in the subsequent experiments (Figure 2). Excessive nitric oxide (NO) production, driven by the interaction of Toll-like receptor 4 (TLR4) with lipopolysaccharides (LPSs), plays a significant role in the progression of inflammatory diseases. Inhibiting the production of inflammatory mediators like NO is a key therapeutic strategy [34,35]. Among the derivatives tested—2′-hydroxy-2,4-dimethoxychalcone (2,4-DMC), 2′-hydroxy-2,5′-dimethoxychalcone (2,5′-DMC), and 2′-hydroxy-2,6′-dimethoxychalcone (2,6′-DMC)—2,6′-DMC exhibited the strongest NO inhibition, prompting further investigation (Figure 3).

Further analysis of 2,6′-DMC showed a significant suppression of prostaglandin E_2_ (PGE_2_) and pro-inflammatory cytokines, including interleukin-1β (IL-1β), interleukin-6 (IL-6), and tumor necrosis factor-α (TNF-α) (Figure 4). The Western blot analysis revealed a decreased expression of inducible nitric oxide synthase (iNOS) and cyclooxygenase-2 (COX-2), enzymes responsible for NO and PGE_2_ production (Figure 5). Additionally, 2,6′-DMC inhibited the phosphorylation of p38 mitogen-activated protein kinase (MAPK), a key regulator of pro-inflammatory cytokine production, indicating the downregulation of the MAPK signaling pathway (Figure 6). The nuclear factor kappa B (NF-κB) pathway, a central regulator of inflammatory responses, was also affected. Treatment with 2,6′-DMC inhibited IκB-α phosphorylation, thereby preventing the nuclear translocation of NF-κB. The significant increase in the cytoplasmic levels of NF-κB p65 confirmed that 2,6′-DMC exerts its anti-inflammatory effects by modulating this critical signaling pathway (Figure 7 and Figure 8).

Melanin is a critical biomolecule responsible for pigmentation in the skin, hair, and eyes, providing protection against ultraviolet (UV) radiation and mitigating oxidative stress. However, excessive melanin production can lead to hyperpigmentation and is associated with conditions such as malignant melanoma and chronic inflammation. Although various inhibitors of melanin synthesis are available, many exhibit cytotoxicity, induce inflammation, or disrupt the skin’s natural defense mechanisms [21,22,23,24,25,26,27,28]. Therefore, there is a need for safe and effective inhibitors that selectively regulate melanin production without adverse effects.

To assess the effects of 2′-hydroxy-2-methoxychalcone derivatives on melanogenesis, MTT assays were used to ensure non-cytotoxic concentrations for the experiments (Figure 9). The results demonstrated that 2,6′-DMC exhibited the most potent inhibitory effects on melanin content and tyrosinase activity compared to 2,4-DMC and 2,5′-DMC (Figure 10 and Figure 11). Further investigations revealed that 2,6′-DMC downregulated the expression of the microphthalmia-associated transcription factor (MITF), which regulates tyrosinase, tyrosinase-related protein 1 (TRP-1), and TRP-2 (Figure 12 and Figure 13). The Wnt/β-catenin signaling pathway, which regulates MITF expression, was also examined. Activation of this pathway leads to the phosphorylation and inactivation of glycogen synthase kinase 3β (GSK3β), releasing β-catenin into the cytoplasm and promoting its nuclear translocation. Treatment with 2,6′-DMC suppressed β-catenin expression and GSK3β phosphorylation, indicating an inhibition of the Wnt/β-catenin pathway (Figure 14).

The phosphoinositide 3-kinase (PI3K)/AKT pathway, which regulates GSK3β activity, was also analyzed. AKT phosphorylates GSK3β at serine 9, inhibiting its function and stabilizing β-catenin [36,37]. This study found that 2,6′-DMC reduced AKT phosphorylation, contributing to decreased GSK3β phosphorylation and β-catenin stabilization (Figure 15). An analysis of the MAPK pathway revealed that extracellular signal-regulated kinase (ERK) promotes MITF degradation and suppresses melanogenesis, while p38 MAPK and c-Jun N-terminal kinase (JNK) enhance MITF expression and melanin production. The results demonstrated that 2,6′-DMC increased ERK phosphorylation while reducing p38 and JNK phosphorylation, supporting its role in suppressing melanin synthesis (Figure 16).

The cAMP/protein kinase A (PKA)/cAMP response element-binding protein (CREB) pathway was also investigated. The binding of an α-melanocyte-stimulating hormone (α-MSH) to a melanocortin 1 receptor (MC1R) leads to cAMP accumulation, PKA activation, and CREB phosphorylation, enhancing MITF transcription [38,39]. This study showed that 2,6′-DMC significantly reduced α-MSH-induced PKA and CREB phosphorylation, further demonstrating its inhibitory effects on melanogenesis (Figure 17).

Additionally, we emphasize that the anti-inflammatory and melanin-inhibitory effects of 2,6′-DMC are mediated through the crosstalk among various interconnected pathways. The NF-κB pathway is closely linked to MAPK signaling, where the activation of p38 MAPK can enhance NF-κB transcriptional activity. By inhibiting p38 MAPK, 2,6′-DMC effectively reduces NF-κB-mediated inflammatory responses. Furthermore, the PI3K/AKT pathway regulates the phosphorylation of GSK3β, a key modulator of the Wnt/β-catenin pathway. 2,6′-DMC suppresses AKT phosphorylation, which in turn decreases GSK3β phosphorylation, leading to the inhibition of β-catenin activity and MITF expression, thereby suppressing melanin production. At the same time, 2,6′-DMC promotes ERK activation, which facilitates MITF degradation, while inhibiting the cAMP/PKA/CREB pathway, thereby reducing MITF transcription. These pathways collectively contribute to the inhibition of melanin synthesis. Moreover, chronic inflammation can influence melanin production through the release of cytokines and oxidative stress. By suppressing inflammatory mediators via the NF-κB and MAPK pathways, 2,6′-DMC indirectly reduces melanin synthesis. These findings highlight the dual anti-inflammatory and anti-melanogenic potential of 2,6′-DMC as a promising therapeutic agent.

Figure 3 and Figure 10 illustrate that 2,6′-DMC significantly suppressed NO production and melanin synthesis levels compared to 2,4-DMC and 2,5′-DMC at equivalent concentrations. Two key factors may explain these observations. First, the electronic and structural properties of methoxy group placement likely contributed. The 2 and 6′ positions contribute to enhanced molecular symmetry and structural stability, which may increase its binding affinity to target proteins. Notably, the methoxy group at position 6′ may optimize active site access, enhancing the inhibitory effects on the signaling pathways involved in NO and melanin production. Furthermore, 2,6′-DMC may interact with molecular targets such as COX-2 and iNOS, which are key enzymes in an inflammatory response. Its structural features might allow it to occupy critical binding pockets within these enzymes, thereby inhibiting their activity. Additionally, 2,6′-DMC could target NF-κB signaling components, such as IκB-α, to prevent the nuclear translocation of p65, as indicated by our experimental results. Second, the increased hydrophobicity due to methoxy groups may improve cellular permeability, allowing 2,6′-DMC to enter cells more effectively and inhibit the expression of enzymes associated with inflammation and melanogenesis. This could include tyrosinase, a key regulator of melanin biosynthesis, and other the transcription factors involved in inflammatory cytokine production [40,41].

The physicochemical properties of 2,6′-DMC are closely linked to the unique characteristics of the chalcone structure, which significantly influence its potential for clinical applications. The chalcone backbone, characterized by an α,β-unsaturated carbonyl group, provides strong reactivity and chemical versatility, enabling a wide range of biological activities. This intrinsic structural feature enhances 2,6′-DMC’s ability to interact selectively with target proteins, effectively inhibiting key enzymes involved in inflammation (e.g., iNOS, COX-2) and melanogenesis (e.g., tyrosinase).

Chalcone derivatives, including 2,6′-DMC, demonstrate an optimal balance between hydrophobicity and hydrophilicity, which improves cellular permeability and ensures efficient delivery to intracellular targets. The methoxy substitutions in 2,6′-DMC further enhance its hydrophobicity, contributing to its stability in biological environments such as the skin. These properties make 2,6′-DMC an effective agent in addressing skin conditions like inflammation and hyperpigmentation, positioning it as a promising candidate for dermatological and cosmetic applications. In clinical evaluations, 2,6′-DMC exhibited low skin irritation, reflecting the physicochemical stability and safety of the chalcone scaffold. This evidence supports its suitability for long-term and repeated use, making it ideal for therapeutic and cosmetic formulations targeting inflammatory skin conditions, hyperpigmentation, and overall skin health enhancement. However, due to the inherent reactivity of the chalcone structure, potential interactions with other active ingredients in combined formulations should be carefully considered. Further studies on these interactions, as well as the long-term safety and efficacy of 2,6′-DMC, are essential to fully realize its clinical potential. Such investigations will help optimize its application and expand its utility in the pharmaceutical and cosmetic industries.

In conclusion, this study demonstrated that 2,6′-DMC effectively inhibits melanin synthesis and inflammatory responses by modulating multiple signaling pathways, including Wnt/β-catenin, PI3K/AKT, MAPK, and cAMP/PKA/CREB. These findings underscore its potential as a therapeutic agent for hyperpigmentation disorders and as a promising component in skin-lightening formulations.

This study utilized murine cell lines, specifically RAW 264.7 macrophages and B16F10 melanoma cells, because these are well-established models in immunological and melanogenesis research, suitable for mechanistic studies and compound screening. While human cell lines are widely available, offer better clinical relevance, and are more commonly used in translational research, murine cell lines provide a cost-effective and reliable platform for preliminary studies. Future research will incorporate human cell models to enhance translational relevance and validate the clinical applicability of 2,6′-DMC.

Additionally, the dual anti-inflammatory and anti-melanogenic properties of 2,6′-DMC highlight its potential as a multifunctional agent for therapeutic and cosmetic applications, warranting further in vivo studies and clinical validation to confirm its efficacy and safety.

## Data Availability

The authors confirm that all the data needed to support this study are presented within the article.
